# Utility of Newborn Dried Blood Spots to Ascertain Seroprevalence of SARS-CoV-2 Antibodies Among Individuals Giving Birth in New York State, November 2019 to November 2021

**DOI:** 10.1001/jamanetworkopen.2022.27995

**Published:** 2022-08-22

**Authors:** Amanda Damjanovic, Linda M. Styer, Katherine Nemeth, Erica Yauney, Jean M. Rock, Rachel Bievenue, Rebecca Hoen, Dylan Ehrbar, Denise M. Kay, Michele Caggana, Monica M. Parker

**Affiliations:** 1 Wadsworth Center, New York State Department of Health, Albany; 2 Department of Biomedical Sciences, School of Public Health, University at Albany, Albany, New York; 3 Bureau of Surveillance and Data Systems, New York State Department of Health, Albany

## Abstract

**Importance:**

Serosurveys can be used to monitor population-level dynamics of COVID-19 and vaccination. Dried blood spots (DBSs) collected from infants contain maternal IgG antibodies and are useful for serosurveys of individuals recently giving birth.

**Objectives:**

To examine SARS-CoV-2 antibody prevalence in pregnant individuals in New York State, identify associations between SARS-CoV-2 antibody status and maternal and infant characteristics, and detect COVID-19 vaccination among this population.

**Design, Setting, and Participants:**

A population-based, repeated cross-sectional study was conducted to detect SARS-CoV-2 nucleocapsid (N) and spike (S) IgG antibodies. Deidentified DBS samples and data submitted to the New York State Newborn Screening Program between November 1, 2019, and November 30, 2021, were analyzed.

**Exposures:**

Prenatal exposure to SARS-CoV-2 antibodies.

**Main Outcomes and Measures:**

The presence of IgG antibodies to SARS-CoV-2 N and S antigens was measured using a microsphere immunoassay. Data were analyzed by geographic region and compared with reported COVID-19 cases and vaccinations among reproductive-aged females (15-44 years of age). Data were stratified by infant birth weight, gestational age, maternal age, and multiple birth status.

**Results:**

Dried blood spot samples from 415 293 infants (median [IQR] age, 1.04 [1.00-1.20] days; 210 805 [51.1%] male) were analyzed for SARS-CoV-2 antibodies. The first known antibody-positive infant in New York State was born on March 29, 2020. SARS-CoV-2 seroprevalence reflected statewide and regional COVID-19 cases among reproductive-aged females in the prevaccine period. From February through November 2021, S seroprevalence was strongly correlated with cumulative vaccinations in each New York State region and in the state overall (*r*_s_ = 0.92-1.00, *P* ≤ .001). S and N seroprevalences were significantly lower in newborns with very low birth weight (720 [14.8%] for S and 138 [2.8%] for N, *P* < .001) and low birth weight (5160 [19.3%] for S and 1233 [4.6%] for N, *P* = .009) compared with newborns with normal birth weight (77 116 [20.1%] for S and 19 872 [5.2%] for N). Lower N and higher S seroprevalences were observed in multiple births (odds ratio [OR], 0.84; 95% CI, 0.75-0.94; *P* = .002 for N and OR, 1.24; 95% CI, 1.18-1.31; *P* < .001 for S) vs single births and for maternal age older than 30 years (OR, 0.87; 95% CI, 0.80-0.94; *P* < .001 for N and OR, 1.17; 95% CI, 1.11-1.23; *P* < .001 for S) vs younger than 20 years.

**Conclusions and Relevance:**

In this study, seroprevalence in newborn DBS samples reflected COVID-19 case fluctuations and vaccinations among reproductive-aged women during the study period. These results demonstrate the utility of using newborn DBS testing to estimate SARS-CoV-2 seroprevalence in pregnant individuals.

## Key Points

**Question:**

Can analysis of newborn dried blood spot (DBS) samples be used to monitor SARS-CoV-2 seroprevalence in infants and individuals giving birth?

**Findings:**

In this repeated cross-sectional study, DBS samples from 415 293 infants born in New York State from November 1, 2019, to November 30, 2021, were analyzed for SARS-CoV-2 antibodies. Statewide and regional seroprevalence data reflect the fluctuations in reported COVID-19 cases and vaccinations among reproductive-aged females during this period in New York State.

**Meaning:**

These findings suggest that antibody testing of newborn DBS samples is an effective way to conduct large-scale monitoring of SARS-CoV-2 seroprevalence among individuals recently giving birth.

## Teaser

This cross-sectional study examines the use of newborn dried blood spot testing in determining the SARS-CoV-2 prevalence among pregnant individuals in New York State.

## Introduction

The first case of SARS-CoV-2 was detected in New York State on February 29, 2020.^1^ Throughout spring 2020, the number of cases increased rapidly, especially in New York City and surrounding regions. A serosurvey of SARS-CoV-2 prevalence in New York State in April 2020 found a cumulative incidence of 14.0% in New York State overall and 22.7% in New York City.^2^ Laboratory-confirmed SARS-CoV-2 diagnoses represented a small proportion of previously infected individuals during this period,^2^ suggesting that the number of SARS-CoV-2 infections had been underestimated.

In response to SARS-CoV-2 infection, most individuals develop antibodies to the spike (S) and nucleocapsid (N) proteins within 1 to 2 weeks, and these antibodies can be measured as an indicator of COVID-19 prevalence in a population.^3,4^ Dried blood spot (DBS) testing has been validated for the measurement of SARS-CoV-2 IgG antibodies against N and S proteins.^5,6,7,8,9,10^ Dried blood spots collected by heel stick from infants born in New York State are submitted to the New York State Newborn Screening Program (NSP), where they are tested for more than 50 congenital disorders. Maternal IgG antibodies can pass through the placenta and provide the newborn with passive immunity to infections after birth.^11^ Detection of IgG antibodies in newborn DBS samples provides information about the mother’s serostatus, and DBS samples from newborns can be used for large-scale surveys of maternal seropositivity.^12,13,14^

The goal of this study was to monitor SARS-CoV-2 antibody prevalence in pregnant individuals by detecting maternal IgG antibodies in newborn DBS samples. We analyzed DBS samples collected from 415 293 newborns born in New York State from November 1, 2019, through November 30, 2021, a period that spans the reported arrival of SARS-CoV-2 in the US and the prevaccine and postvaccine phases in New York State. We used a multiplex SARS-CoV-2 microsphere immunoassay that was validated for DBS testing^10^ and separately detects IgG antibodies to SARS-CoV-2 N and S antigens. Measuring N and S antibodies independently is useful because both antibodies are known to wane over time, with mean half-lives of 85 days for N and 180 days for S.^15,16^ In addition, all COVID-19 vaccines currently available in the US produce antibodies to the S antigen only. We aimed to examine SARS-CoV-2 antibody prevalence in pregnant individuals in New York State, monitor seroprevalence over time among individuals recently giving birth by New York State region, identify associations between SARS-CoV-2 antibody status and maternal and infant characteristics, and detect COVID-19 vaccination among this population.

## Methods

### Specimens

This cross-sectional study used DBS samples from infants born in New York State from November 1, 2019, through November 30, 2021. Samples were submitted to the NSP and punched (3.2 mm) into 96-well plates, blinded, and stored at 2 to 8 °C until tested. A total of 416 278 DBS samples met the study inclusion criteria. Of these, 985 (0.2%) were unsatisfactory for testing, leaving a study sample of 415 293 DBS samples. This study was approved by the New York State Department of Health Institutional Review Board, which waived informed consent because only deidentified residual specimens were used. This study followed the Strengthening the Reporting of Observational Studies in Epidemiology (STROBE) reporting guideline.

### Assay Procedure

Magplex-C microspheres (Luminex Corp) were coupled with SARS-CoV-2 Spike subunit S1 (S) (Sino Biological) and nucleocapsid (N) (Native Antigen) antigens. SARS-CoV-2 IgG microsphere immunoassay testing was conducted as previously described,^10^ except that Hamilton Microlab Star liquid handling systems were used for DBS elution, microsphere and secondary antibody addition, and incubations. The mean median fluorescence intensity (MFI) of at least 92 SARS-CoV-2 antibody–negative DBS samples was used to set cutoffs for each bead set. Multiple bead lots were used, and a mean of the cutoffs for each antigen was used to determine reactivity. Median fluorescence intensity values less than the mean MFI + 6 SDs were nonreactive, and values greater than the mean MFI + 6 SDs were reactive. Index values were calculated by dividing the sample MFI by the cutoff for each antigen; indexes greater than 1.0 indicated a reactive result.

### Statistical Analysis

Data extracted from the NSP database included infant sex, birth weight, gestational age, birth date, transfusion status, twin status, DBS collection date, birth hospital, maternal birth date, county, and zip code. Data on race and ethnicity could not be reported because the NSP blood collection form does not include this information. A blinded study identifier was assigned to each specimen, and a linking identifier was used to identify repeat samples. Samples excluded were repeats from the same infant, collected more than 30 days after birth, from infants whose parents opted out of public health research, from infants who had received transfusions, or from infants of mothers residing outside New York State. Only first-born infants of multiple gestation births were included. Cleaned data were collapsed, and the final data set included age at blood collection; week, month, and year of birth; sex; gestational age; birth weight; maternal age; maternal county; and antibody results. Data were missing for less than 2.02% of samples, and missing data were ignored in the analysis via pairwise deletion. Correlations were assessed by the Spearman correlation coefficient in GraphPad Prism (GraphPad Software). Logistic regression was used to test for association between S and N antibody reactivity and demographic factors using R software, version 4.0.2 (R Foundation for Statistical Computing). To assess for confounding variables among demographic factors, multicollinearity tests were performed. An α of .05 was considered statistically significant.

Data for reproductive-aged females (15-44 years of age) included total population per county in New York State in 2020 obtained from the US Census Bureau, reported COVID-19–positive cases from March 2020 through November 2021 from the Bureau of Surveillance and Data Systems of the New York State Department of Health, and completed vaccination series through November 2021 from the Citywide Immunization Registry and New York State Immunization Information System. Data were aggregated by county and deduplicated. Weeks are numbered by calendar year, with week 1 beginning on January 1.

## Results

**Figure 1.**
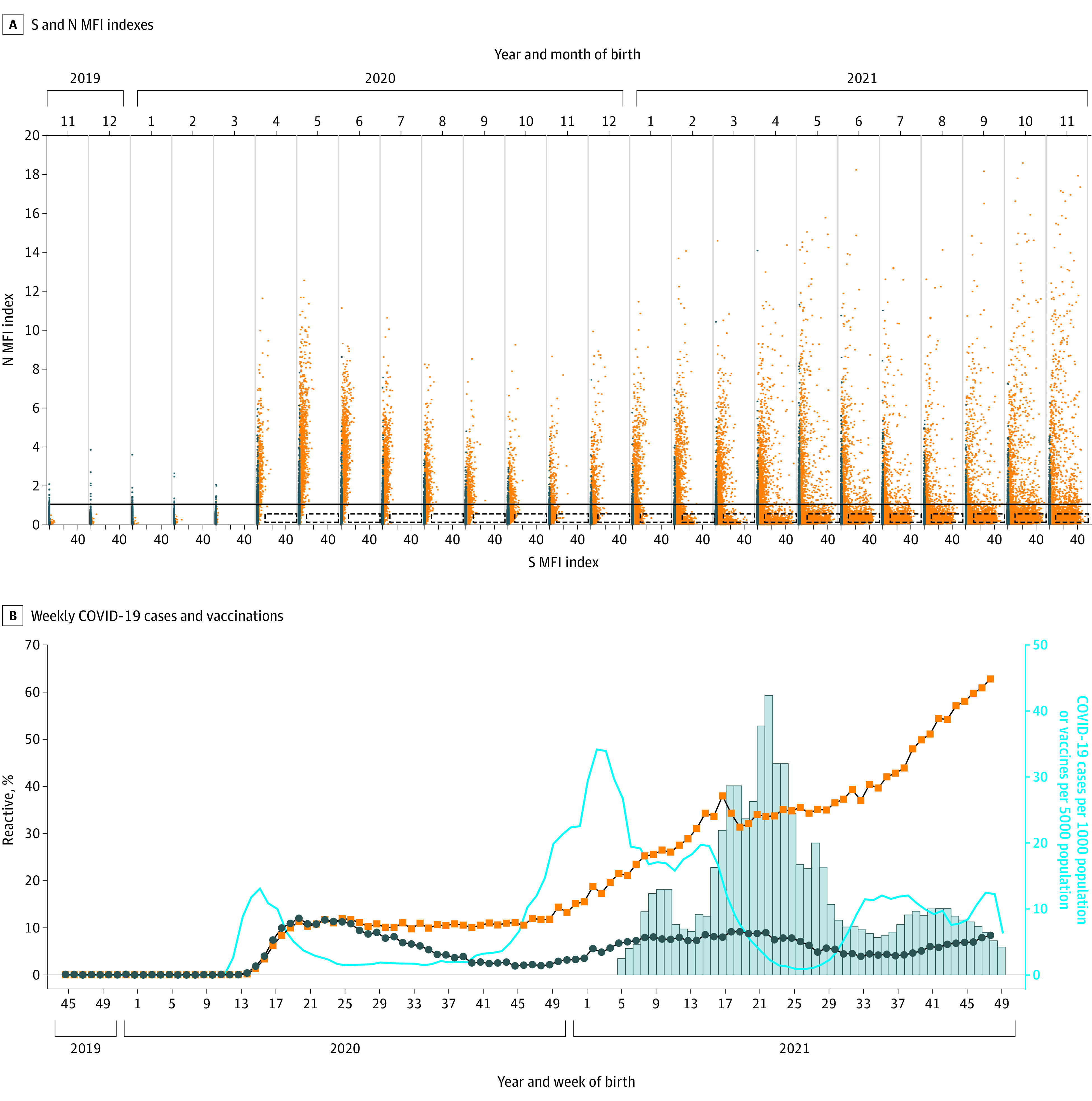
SARS-CoV-2 Spike S1 and Nucleocapsid Antibodies in Dried Blood Spot (DBS) Samples Collected From November 2019 Through November 2021 A, Spike (S) and nucleocapsid (N) median fluorescence intensity (MFI) indexes for DBS samples collected from infants born from November 2019 through November 2021. Indexes above 1.0 are considered reactive. The N index reactive cutoff is indicated by a solid line. The DBS samples with S indexes of 1.0 or higher are orange and less than 1.0 are gray. The boxes with dashed lines indicate samples with high S (>7) and low N (<0.5) indexes, indicative of recent vaccination. On the x-axis, for each month, the 2 unlabeled tick marks represent S MFI index values of 0 (left) and 20 (right). B, Weekly COVID-19 cases (blue line) and vaccinations (bars) among females aged 15 to 44 years in New York State and percentage of DBS samples reactive for S (orange squares) and N (blue circles) antibodies by week of birth. Week of birth ranges from November 3, 2019 (2019 week 45) through November 29, 2021 (2021 week 49).

 Dried blood spots from 415 293 infants (median [IQR] age, 1.04 [1.00-1.20] days; 210 805 [51.1%] male and 201 814 [48.9%] female of 412 619 infants with reported sex) were analyzed for SARS-CoV-2 antibodies. To verify assay specificity, we tested samples collected from infants born from November 1, 2019, through December 31, 2019, because it is unlikely that SARS-CoV-2 antibodies would be present in infants born during this period. Of 34 231 DBS samples, 39 (0.10%) were reactive for S antibodies, 28 (0.08%) were reactive for N antibodies, and none were reactive for both N and S. Index values were low, with maximum values of 8.48 for S and 3.84 for N (Figure 1A). Although each individual antigen displayed high specificity (≥99.9%), requiring both S and N reactivity increased specificity to 100%. Using this criterion, the first samples reactive for both S and N antibodies were from 6 infants born between March 29 and April 4, 2020 (week 14 of 2020) (Figure 1B). Individuals giving birth to these infants resided in either New York City or a nearby downstate county, consistent with the locations of early reported cases in New York State.^2^

### Weekly Statewide SARS-CoV-2 Antibody Results

We next examined antibody reactivity during the first wave of COVID-19, from March to June 2020. Data on pregnancy status of confirmed COVID-19 cases were not available; however, New York State surveillance data on COVID-19 cases reported for reproductive-aged females were accessible (Figure 1B). COVID-19 cases among this population were first reported at week 10 of 2020, and cases peaked at week 15 of 2020. Seroprevalence in newborns increased rapidly from week 14 of 2020, reaching a peak of 12.1% for N at week 20 of 2020 and 12.0% for S at week 25 of 2020 (Figure 1B), 5 to 10 weeks after peak cases among reproductive-aged females. N seroprevalence decreased from weeks 26 to 45 of 2020, whereas S seroprevalence decreased slightly and remained stable.

The second wave of COVID-19 cases started in week 41 of 2020; cases continued to increase through week 2 of 2021 (Figure 1B). Newborn seroprevalence increased starting at week 50 of 2020 and continued through week 19 of 2021 for N, 17 weeks after second-wave cases peaked. The increase in N seroprevalence after the second wave of COVID-19 cases was more gradual than after the first wave.

S seroprevalence and index values continued to increase from December 2020 through November 2021, whereas N seroprevalence peaked at week 19 of 2021. A high level of S antibodies without N antibody reactivity is a signature of recent COVID-19 vaccination because all currently approved COVID-19 vaccines induce antibodies to S only. On the basis of data from testing prevaccinated and postvaccinated individuals (L.M.S., unpublished data, December 20, 2021), we categorized samples as presumed vaccinated if the index for S was greater than 7.0 and the index for N was less than 0.5. These criteria will not identify individuals as vaccinated if they were previously infected because the N index would likely be higher than 0.5. Samples with high S and low N indexes increased beginning in January 2021 and continued through November 2021 (Figure 1A), consistent with an increase in vaccinations in eligible females aged 15 to 44 years in New York State.

### Monthly SARS-CoV-2 Antibody Results by New York State Region

**Figure 2.**
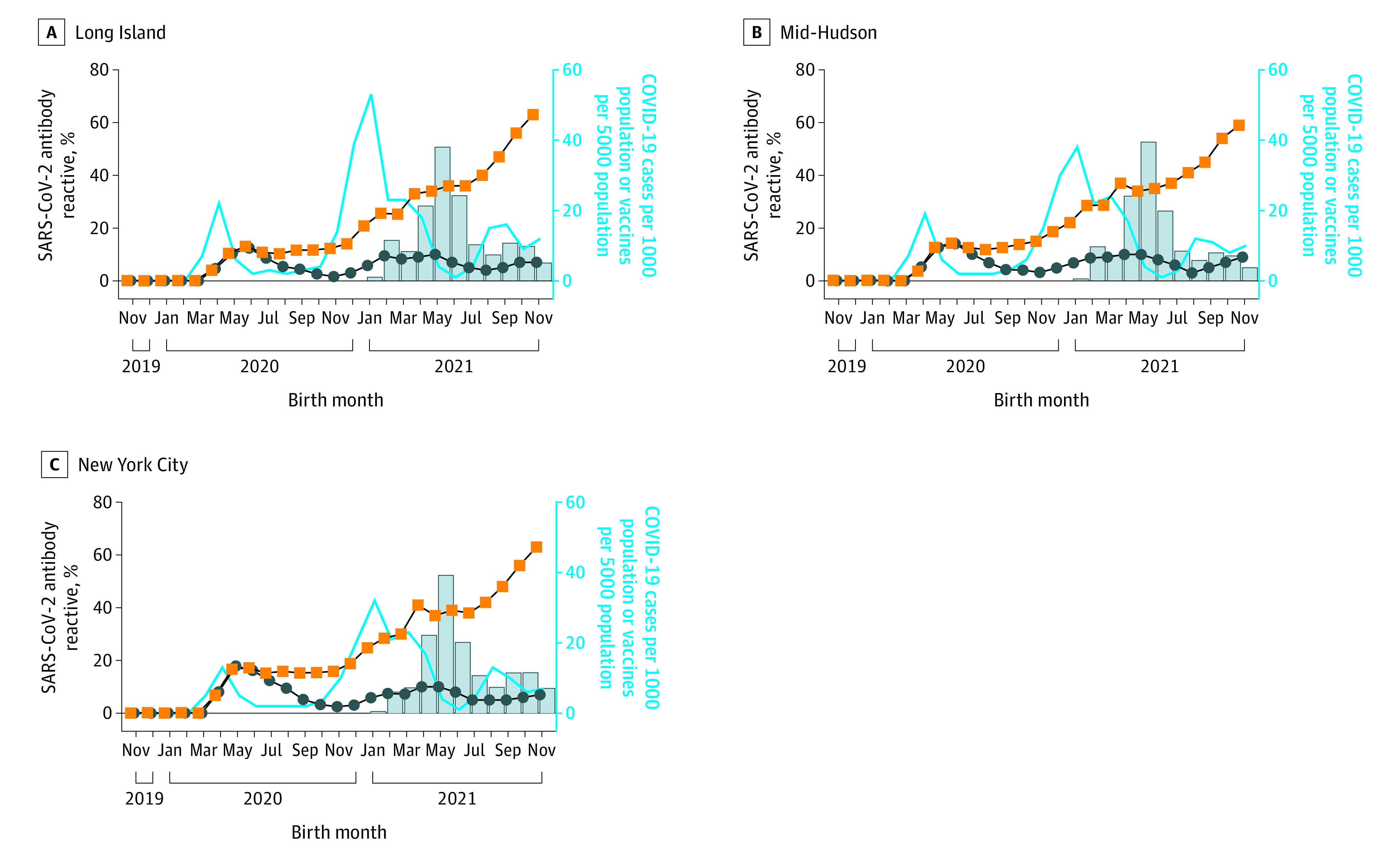
Newborn SARS-CoV-2 Antibody Reactivity and COVID-19 Cases and Vaccinations in Reproductive-Aged Females in New York State Regions With High COVID-19 Incidence Percentage of SARS-CoV-2 spike (orange squares) or nucleocapsid (blue circles) antibody reactivity in eluted dried blood spot samples from newborns and COVID-19 cases (blue line) and vaccinations (bars) in females aged 15 to 44 years.

We grouped New York State counties into 10 regions (eFigure in the Supplement) and analyzed SARS-CoV-2 seroprevalence in infants and those with COVID-19 and vaccinations among reproductive-aged females by region (Table 1). In the 3 highest seroprevalence regions, cases spiked in April 2020 and newborn seroprevalence peaked in May and June, after which N seroprevalence decreased, whereas S seroprevalence remained stable (Figure 2). COVID-19 cases spiked again in November 2020 followed by a gradual increase in N seroprevalence that leveled off and decreased in June 2021. A third wave of cases in July 2021 was followed by an increase in N seroprevalence in September 2021. S seroprevalence increased more rapidly and corresponded with increases in COVID-19 vaccinations beginning in January 2021 (Figure 2). From November 2019 through January 2021, there was a moderate positive correlation between COVID-19 cases and S seroprevalence (*r*_s_ = 0.64-0.66, *P* = .01) but no correlation with N seroprevalence. As vaccinations increased from February through November 2021, a strong positive correlation between cumulative vaccinations in reproductive-age females and S seroprevalence was observed (*r*_s_ = 0.92-0.98, *P* ≤ .001).

**Table 1.** Regional SARS-CoV-2 Cases, Vaccinations, and Newborn Antibody Reactivity in New York State From November 2019 to November 2021

Region	Total No. (%) of births^a^	No. (%) of births		Total No. of females aged 15-44 y	No. (%) of females aged 15-44 y	
		S antibody reactive	N antibody reactive		COVID-19 cases	Vaccinated
New York City	189 476 (46.6)	43 113 (22.8)	11 609 (6.1)	1 846 594	377 000 (20.4)	1 431 234 (77.5)
Long Island	56 872 (14.0)	11 958 (21.1)	3022 (5.3)	510 697	144 009 (28.2)	406 364 (79.6)
Mid-Hudson	47 488 (11.7)	10 323 (21.7)	2863 (6.0)	423 715	103 345 (24.4)	308 980 (72.9)
Western	26 808 (6.6)	3983 (14.9)	905 (3.4)	256 971	54 698 (21.3)	167 130 (65.0)
Finger Lakes	23 538 (5.8)	3444 (14.6)	750 (3.2)	225 901	43 910 (19.4)	147 900 (65.5)
Capital	19 524 (4.8)	2813 (14.4)	485 (2.5)	203 569	33 966 (16.7)	142 680 (70.1)
Central	15 112 (3.7)	1992 (13.2)	448 (3.0)	149 106	28 466 (19.1)	99 749 (66.9)
Southern	10 846 (2.7)	1292 (11.9)	274 (2.5)	119 673	22 083 (18.5)	72 882 (60.9)
Mohawk	9185 (2.3)	1024 (11.1)	278 (3.0)	76 913	18 146 (23.6)	44 219 (57.5)
North Country	8059 (2.3)	842 (10.4)	174 (2.2)	74 660	12 089 (16.2)	44 685 (59.9)
Total	406 908 (100)	80 784 (19.9)	20 808 (5.1)	3 887 799	837 712 (21.5)	2 865 823 (73.7)

^a^ A total of 8385 newborns with unknown counties were excluded.

**Figure 3.**
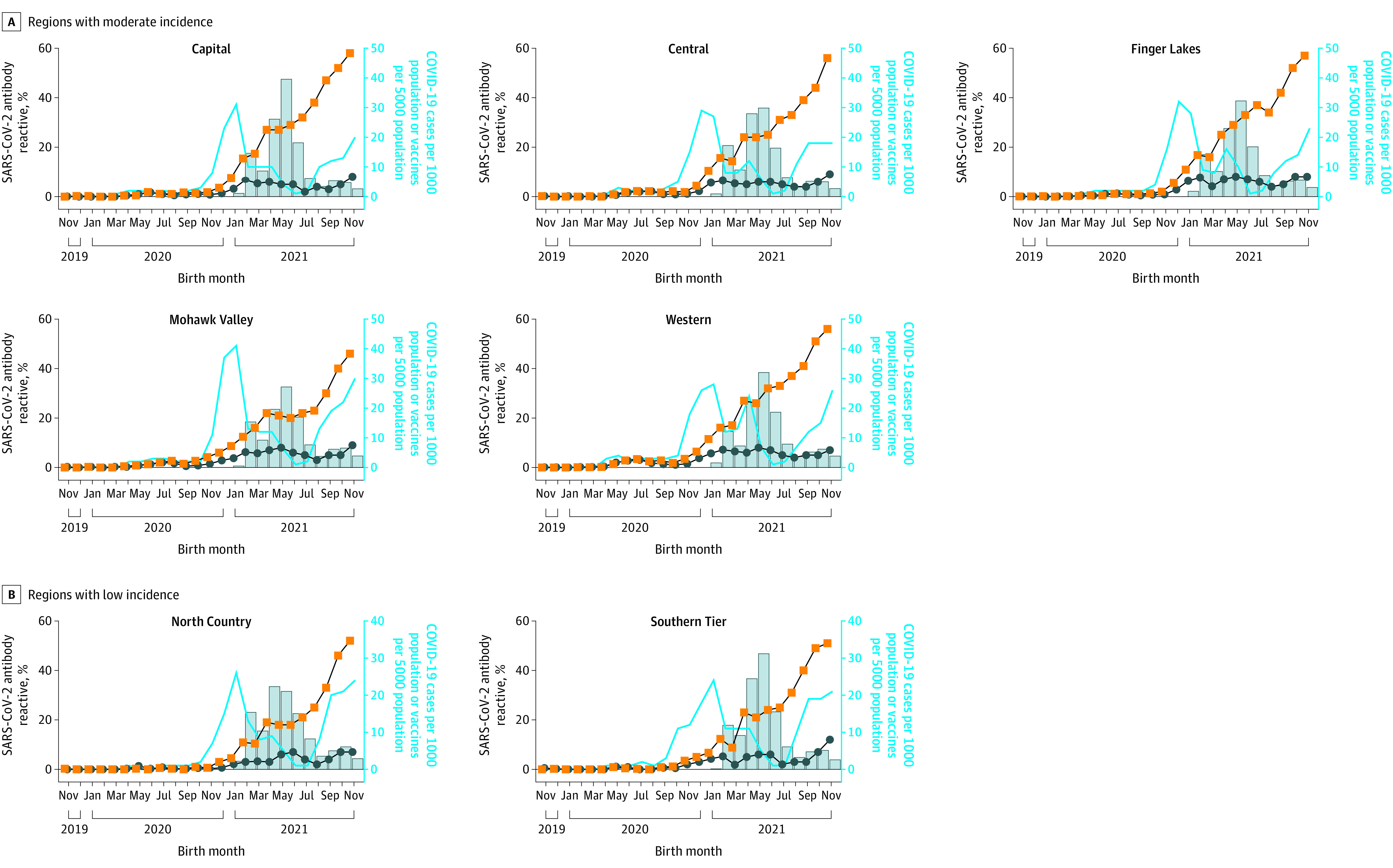
Newborn SARS-CoV-2 Antibody Reactivity and COVID-19 Cases and Vaccinations in Reproductive-Aged Females in New York State Regions With Moderate and Low COVID-19 Incidence Percentage of SARS-CoV-2 spike (orange squares) or nucleocapsid (blue circles) antibody reactivity in eluted dried blood spot samples from newborns and COVID-19 cases (blue line) and vaccinations (bars) in females aged 15 to 44 years.

Five regions had moderate case numbers during spring 2020 and moderate seroprevalence (Figure 3A). Cases surged during the fall, peaking in December 2020 and January 2021, followed by a rapid increase in N and S seroprevalence (Figure 3A). From January through November 2021, N seroprevalence reflected fluctuations in COVID-19 cases, whereas S seroprevalence increased continuously, reflecting increases in vaccination. From November 2019 through January 2021, case counts correlated strongly with S (*r*_s_ = 0.80-0.96, *P* ≤ .001) and N (*r*_s_ = 0.77-0.91, *P* ≤ .001) seroprevalence. From February through November 2021, a strong positive correlation was found between cumulative vaccinations and S seroprevalence (*r*_s_ = 0.94-1.00, *P* ≤ .001).

Two regions had low COVID-19 case numbers until October 2020 (Figure 3B). Seroprevalence increased from November and December 2020 through May 2021. A second increase in N seroprevalence began in September 2021 following a surge of new COVID-19 cases. S seroprevalence continued to increase, corresponding with an increase in vaccinations. From November 2019 through January 2021, a strong positive correlation was found between COVID-19 cases and S seroprevalence (*r*_s_ = 0.77, *P* = .002 and *r*_s_ = 0.87, *P* < .001) and a moderate correlation with N seroprevalence (*r_s_* = 0.66, *P* = .009 and *r*_s_ = 0.68, *P* = .007) in these 2 regions. From February through November 2021, a strong positive correlation was found between cumulative vaccinations and S seroprevalence (*r*_s_ = 0.96, *P* ≤ .001 and *r*_s_ = 0.98, *P* ≤ .001). Therefore, S seroprevalence was strongly correlated with cumulative vaccinations in each of 10 New York State regions and in the state overall (*r*_s_ = 0.98, *P* < .001).

### Demographic Analysis

SARS-CoV-2 N and S seroprevalence were analyzed by multivariate logistic regression against maternal and newborn characteristics (Table 2). S seroprevalence was significantly higher in females (40 657 [20.1%]) compared with males (41 862 [19.9%], *P* = .01), whereas N seroprevalence did not differ by sex (Table 2). Multiple births were associated with higher S (2062 [22.5%], *P* < .001) and lower N (368 [4.0%], *P* = .002) seroprevalence compared with single births (80 921 [19.9%] for S seroprevalence and 20 871 [5.1%] for N seroprevalence).

**Table 2.** Analysis of Demographic Characteristics and SARS-CoV-2 N and S Antibody Reactivity Using a Logistic Regression Model^a^

Characteristic	Total No. of infants (N = 415 293)	S antibody reactive, No. (%) (n = 83 035)	Odds ratio (95% CI)	*P* value	N antibody reactive, No. (%) (n = 21 254)	Odds ratio (95% CI)	*P* value
Maternal age, y							
<20	11 138	2104 (18.9)	1 [Reference]	NA	618 (5.5)	1 [Reference]	NA
20-30	186 627	34 459 (18.5)	0.97 (0.93-1.02)	.29	10 126 (5.4)	0.98 (0.90-1.07)	.61
>30	214 553	45 806 (21.3)	1.17 (1.11-1.23)	<.001	10 358 (4.8)	0.87 (0.80-0.94)	<.001
Sex							
Male	210 805	41 862 (19.9)	1 [Reference]	NA	10 737 (5.1)	1 [Reference]	NA
Female	201 814	40 657 (20.1)	1.02 (1.00-1.04)	.01	10 374 (5.1)	1.01 (0.99-1.04)	.33
Gestational age, wk							
>36	375 166	75 178 (20.0)	1 [Reference]	NA	19 322 (5.2)	1 [Reference]	NA
20-36	36 311	6908 (19.0)	0.97 (0.94-1.01)	.10	1628 (4.5)	0.98 (0.92-1.04)	.52
Birth weight, g							
>2499	383 132	77 116 (20.1)	1 [Reference]	NA	19 872 (5.2)	1 [Reference]	NA
1500-2499	26 759	5160 (19.3)	0.93 (0.90-0.97)	<.001	1233 (4.6)	0.91 (0.85-0.98)	.009
<1500	4860	720 (14.8)	0.67 (0.61-0.73)	<.001	138 (2.8)	0.55 (0.45-0.65)	<.001
Twin status							
Single births	405 944	80 921 (19.9)	1 [Reference]	NA	20 871 (5.1)	1 [Reference]	NA
Multiple births	9147	2062 (22.5)	1.24 (1.18-1.31)	<.001	368 (4.0)	0.84 (0.75-0.94)	.002

Abbreviation: NA, not applicable.

^a^ Numbers reported are not equal to the total stated population in the table because data for various characteristics were missing for some individuals.

S and N seroprevalences were significantly lower in newborns with very low birth weight (720 [14.8%] for S seroprevalence and 138 [2.8%] for N seroprevalence, *P* < .001) and low birth weight (5160 [19.3%] for S seroprevalence and 1233 [4.6%] for N seroprevalence, *P* = .009) compared with mean birth weight (77 116 [20.1%] for S seroprevalence and 19 872 [5.2%] for N seroprevalence) (Table 2). Premature (≤36 weeks’ gestation) newborns had lower S seroprevalence (6908 [19.0%], *P* = .10) and N seroprevalence (1628 [4.5%], *P* = .52) than full-term newborns (75 178 [20.0%] for S seroprevalence and 19 872 [5.2%] for N seroprevalence), but these differences were not significant (Table 2). Maternal age older than 30 years was associated with higher S seroprevalence (45 806 [21.3%], *P* < .001) and lower N seroprevalence (10 358 [4.8%], *P* < .001) compared with those younger than 20 years (2104 [18.9%] for S seroprevalence and 618 [5.5%] for N seroprevalence) (Table 2). Variance inflation factors (VIFs) for gestational age (VIF = 1.5), birth weight (VIF = 1.5), and twin status (VIF = 1.1) suggest that multicollinearity is below problematic levels for the models.

## Discussion

This study found that antibody reactivity in newborn DBS samples can be used to estimate maternal seroprevalence at delivery and model COVID-19 cases in a population, as previously reported.^12,17^ The first newborns with SARS-CoV-2 antibodies in New York State were born in or near New York City between March 29 and April 4, 2020. Given that it takes 13 to 15 days to develop IgG antibodies to SARS-CoV-2 and additional time is needed for active transplacental transfer, we estimate that infection occurred at least 2 to 3 weeks before delivery, or approximately March 7, 2020.^12,18^ This estimate is consistent with detection of SARS-CoV-2 antibodies in obstetrics-gynecology patient samples collected during the first week of March 2020.^19^ The first COVID-19 case in New York State was identified in New York City on February 29, 2020,^1^ and the first polymerase chain reaction–positive pregnant individual in New York City was identified on March 13, 2020.^20^ More than 1 in 8 asymptomatic pregnant individuals giving birth in New York City from March 22 to April 4, 2020, were reported to have tested positive for SARS-CoV-2 by polymerase chain reaction testing.^21^ These reports^19,20,21^ support our findings and suggest that the individuals giving birth to the first seropositive infants in our study were likely among the earliest SARS-CoV-2 infections in New York State.

By late March 2020, New York City was a COVID-19 epicenter. From the middle of March through April 1, 2020, SARS-CoV-2 seroprevalence in the NYC metropolitan area was estimated at 6.9% and increased to 23.6% between May and July 2020.^22,23^ In April 2020, the cumulative incidence of COVID-19 among females in New York City was estimated at 22.1%.^2^ We found an S seroprevalence of 6.8% in April 2020 and 17.2% in June 2020 in infants born to New York City residents, suggesting that incidence rates in pregnant women during spring 2020 were similar to those of the general New York City population.

We observed that SARS-CoV-2 seroprevalence in newborns reflected the temporal dynamics of COVID-19 infections reported among reproductive-aged females in New York State overall and in each of 10 regions. In general, we observed an increase in N and S seroprevalence in newborns approximately 1 to 2 months after an increase in reported COVID-19 cases. This pattern was particularly apparent in the regional data because incident cases increased at different times during the study period. Reported cases decreased sharply from January to March 2021 throughout New York State, and N seroprevalence leveled off in most regions in February and March 2021. In contrast, S antibody reactivity continued to increase from January 2021 through November 2021, correlating with COVID-19 vaccinations.

Newborn samples with high levels of spike antibodies and low levels of nonreactive nucleocapsid antibodies is indicative of recent maternal COVID-19 vaccination. COVID-19 vaccines became available in December 2020 to specific populations, including health care and long-term care workers. Although pregnant women were not initially prioritized for vaccination, many got vaccinated because of other risk factors.^24^ Previous studies^25,26^ reported higher S antibodies in neonates born to vaccinated individuals compared with previously infected individuals, implying that by analyzing relative ratios of N and S antibodies, we can make predictions regarding the vaccination status of pregnant individuals.

We found lower N and S seroprevalence in infants with low and very low birth weight, lower N seroprevalence in multiple births, and higher S seroprevalence in female births. Egerup et al^27^ found a similar association between SARS-CoV-2 antibodies and newborn birth weight, but it was nonsignificant when corrected for gestational age and infant’s length at birth.^27^ Previous studies report a reduced transplacental transfer of IgG in placental pathologies, prematurity, low-birth-weight infants, and male fetuses^28,29,30,31^ and suggest SARS-CoV-2 infection may be a risk factor for prematurity.^32^ Specific to SARS-CoV-2, transplacental transfer occurs at a higher efficiency when infection is more than 2 months before delivery but decreases if infection occurs in the third trimester.^33,34,35^ The timing of COVID-19 infection during pregnancy and the duration of transplacental immunity may be useful to explain differences in SARS CoV-2 antibody reactivity with gestational age.

We found lower N seroprevalence with maternal age above 30 years, consistent with others who reported higher frequency of COVID-19 among younger (≤24 years) women giving birth.^36^ We also found higher S seroprevalence associated with maternal age above 30 years and multiple births. Higher and more persistent SARS-CoV-2 S antibody levels in maternal serum and cord blood have been reported in vaccinated women compared with patients who have recovered from COVID-19.^17^ Our results suggest that older pregnant individuals and those with higher-risk pregnancies had less exposure to SARS-CoV-2, possibly through greater adherence to COVID-19 precautions, including vaccination.

### Strengths and Limitations

Strengths of our study include the magnitude of data providing a longitudinal assessment of SARS-CoV-2 seroprevalence over a time frame that spans the beginning of the pandemic through vaccine implementation. We present temporal and geospatial data on N and S seroprevalence in newborns for 10 regions of New York State, with strong positive correlations to COVID-19 cases and vaccinations in reproductive-aged females.

Our study also has several limitations. We were limited to maternal and newborn data submitted on the NSP blood collection form, which did not include race and ethnicity. We also did not have access to maternal COVID-19 diagnosis or maternal vaccination data for the newborns in our study. We tested most infants born in New York State from November 2019 through November 2021 and thus can accurately assess seroprevalence among individuals recently giving birth in New York State, but it is unknown whether our findings can be used to estimate seroprevalence for other populations. By measuring antibodies in newborns, we cannot determine the timing of the SARS-CoV-2 infection or vaccination, only that maternal antibodies were present before giving birth. In addition, SARS-CoV-2 is an acute infection that resolves, and antibodies are expected to decrease and potentially become undetectable over time. Although this will allow for detection of more recent infections and/or vaccination, antibody data cannot be used to determine the total number of people who were infected with SARS-CoV-2.

## Conclusions

This study demonstrates the utility of using residual newborn DBS samples to estimate SARS-CoV-2 seroprevalence in pregnant individuals, without the need to collect new samples. Our high-throughput DBS immunoassay provides an efficient method for monitoring the spread of SARS-CoV-2 and the uptake of vaccines and could be adopted for other emerging infectious diseases.

## Notes

Supplement. **eFigure.** Map of 10 New York State Regions Used for SARS-CoV-2 Seroprevalence Analysis
