# Supplementary material for: Utility of Newborn Dried Blood Spots to Ascertain Seroprevalence of SARS-CoV-2 Antibodies Among Individuals Giving Birth in New York State, November 2019 to November 2021
Source: JAMA Netw Open. 2022 Aug 22;5(8):e2227995. doi: 10.1001/jamanetworkopen.2022.27995 (PMC9396364; doi:10.1001/jamanetworkopen.2022.27995)
Supplement: Supplement. — eFigure. Map of 10 New York State Regions Used for SARS-CoV-2 Seroprevalence Analysis [file jamanetwopen-e2227995-s001.pdf]

Supplementary Online Content

Damjanovic A, Styer LM, Nemeth K, et al. Utility of newborn dried blood spots to ascertain seroprevalence of SARS-CoV-2 antibodies among individuals giving birth in New York State, November 2019 to November 2021. *JAMA Netw Open*. 2022;5(8):e2227995.
doi:10.1001/jamanetworkopen.2022.27995

eFigure. Map of 10 New York State Regions Used for SARS-CoV-2 Seroprevalence Analysis

This supplementary material has been provided by the authors to give readers additional information about their work.

© 2022 Damjanovic A et al. *JAMA Network Open.*

eFigure. Map of 10 New York State Regions Used for SARS-CoV-2 Seroprevalence Analysis

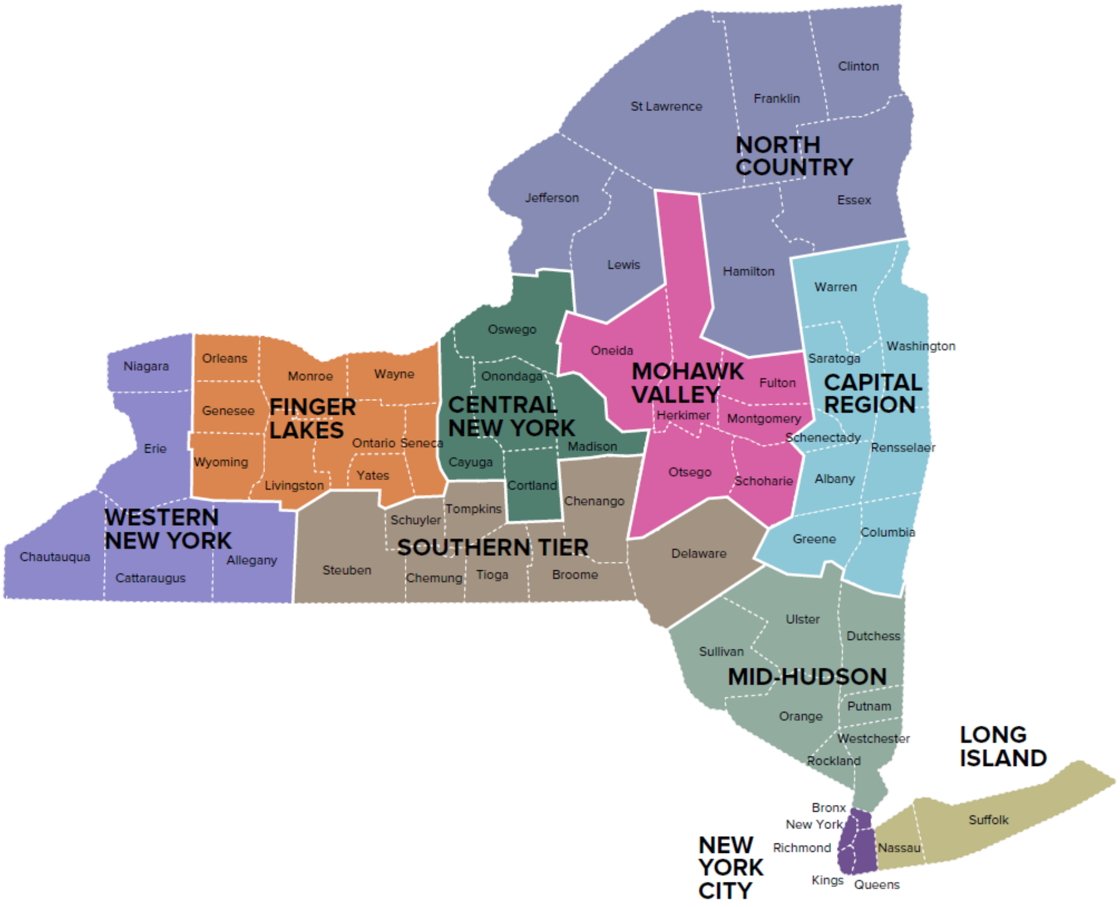

© 2022 Damjanovic A et al. *JAMA Network Open.*
